# The serum 25(OH)D level and hand grip strength for fall risk assessment among osteoporotic elderly Japanese women

**DOI:** 10.1007/s11657-021-00901-0

**Published:** 2021-02-24

**Authors:** Takashi Nagai, Ichiro Okano, Koji Ishikawa, Takuma Kuroda, Yusuke Oshita, Koki Tsuchiya, Soji Tani, Hiroki Okamura, Keizo Sakamoto, Katsunori Inagaki

**Affiliations:** Department of Orthopaedic Surgery, Showa University School of Medicine, 1-5-8 Hatanodai, Sinagaka-ku, Tokyo, 142-8666 Japan

**Keywords:** Osteoporosis, Vitamin D, 25(OH)D, Fall score, Grip strength, Sarcopenia

## Abstract

***Summary*:**

We investigated the relationship between serum 25(OH)D levels, grip strength, and fall score in elderly osteoporotic women for fall risk assessment. Both low serum 25(OH)D and low grip strength were independently associated with increased fall risk. The serum 25(OH)D cutoff specific to increased fall risk was 14 mg/dL (35 nmol/L).

**Purpose:**

This study aimed to establish a cutoff value of serum 25-hydroxyvitamin D (25(OH)D) for fall assessment and investigate the relationship between serum 25(OH)D, grip strength, and fall score adjusted for age in osteoporotic elderly Japanese women.

**Methods:**

This is a cross-sectional study utilizing collected data of osteoporotic elderly (age ≥65 years) female patients. A questionnaire for fall risk assessment was used, in which a score ≥ 6 was determined as increased fall risk. Serum 25(OH)D levels and grip strength were measured, and the cutoff points were calculated by receiver operating curve (ROC) analysis. Logistic regression analysis with age adjustment was conducted for potential risk factors for fall.

**Results:**

After applying eligibility criteria, finally, 349 patients were enrolled. The median patient age was 77.0 years, and the mean serum 25(OH)D level was 15.6 ng/mL (36 nmol/L). Based on the ROC analysis, we defined the cutoff values of serum 25(OH)D level and grip strength as 14 ng/mL (35 nmol/L) and 15 kg, respectively. A multivariate analysis adjusted for age was conducted. Low serum 25(OH)D level and grip strength were independent risk factors for ≥6 fall risk scores.

**Conclusion:**

Both low serum 25(OH)D level and low grip strength were independently associated with increased fall risk score in osteoporotic elderly women. The appropriate serum 25(OH)D cutoff specific to the increased fall risk group in this population was 14 mg/dL (35 nmol/L). These findings might be used for the identification of patients with high fall risks. These results should be confirmed in other patient groups.

## Introduction

Vitamins D_2_ and D_3_ are synthesized from provitamins D_2_ and D_3_, respectively, by absorption of ultraviolet rays in the skin. Additionally, vitamin D is orally ingested from food. Once both vitamins D are absorbed, the first and 25th positions of vitamin D are hydroxylated in the liver and kidney to form 1,25(OH)_2_D_3_, which has active effects in various organ systems [1, 2]. Vitamin D is an essential factor for musculoskeletal organs. It has been reported that an increase in bone density is facilitated by a sufficient supply of active vitamin D_3_ [3–5]. Moreover, its relationship with muscle has been reported; for example, atrophy of type II muscle fibers has been reported in sarcopenic patients with vitamin D deficiency [6]. Furthermore, vitamin D receptors are found in the central nervous system, and it has been suggested that vitamin D and its derivative forms have a neuroprotective effect [7, 8].

Regarding the definitions of vitamin D insufficiency and deficiency, the American Endocrine Society Guidelines [9] and the International Osteoporosis Foundation Guidelines [10] state that vitamin D is considered sufficient if the 25(OH)D level is ≥30 ng/mL, insufficient if it is 20–30 ng/mL, and deficient if it is <20 ng/mL. Among community-dwelling elderly people, the serum concentration of 25(OH)D decreases substantially with age and is generally lower in women than in men [11]. With regard to the association between 25(OH)D and fall, vitamin D supplementation with 25(OH)D serum level above 24 ng/mL (60 nmol/L) was recommended for fall prevention [12, 13]. A study showed that 25(OH)D deficiency (<20 ng/mL) has been considered a risk factor for falls and that once the 25(OH)D falls below 20 ng/mL, the frequency of falls significantly increases [14].

Since 2018, serum 25(OH)D testing for osteoporotic patients has been covered by the national health insurance in Japan and has been widely used in osteoporosis care as well as fall risk assessment. However, the cutoff values were established based on studies for fracture prevention. In the clinical setting, there are only a limited number of osteoporotic elderly female patients who demonstrate 25(OH)D levels above 20 ng/mL. Although it is well known that serum 25(OH)D level is associated with fall risk, it is difficult to utilize this value for further risk stratification in this patient population**.**

Additionally, recent studies have demonstrated that grip strength, as a marker of sarcopenia, is related to fall risk [15, 16]. However, considering the potential effect of vitamin D on non-muscle organ systems, the serum 25(OH)D measurement might have an additional predictive value for fall risk assessment.

In this cross-sectional study, we set two objectives: (1) to establish another cutoff value of serum 25(OH)D concentration, specifically for further stratification of patients with increased fall risks in osteoporotic elderly women, in whom the baseline fall risk is higher and the potential impact of fall is likely greater than the general population, given the increased risk of subsequent fractures, and (2) to investigate the relationship between serum 25(OH)D levels, grip strength, and fall score [17], adjusted for age in this population.

## Methods

This study complies with the guidelines of the Declaration of Helsinki regarding research involving human subjects. This study was reviewed and approved by the Ethics Committee on Research Involving Human Subjects of the Showa University Graduate School of Medicine in the interest of ethical considerations and protection of personal information (approval no. 3014).

### Study design and subjects

This study is a cross-sectional study using data, including fall score and grip strength, collected at the first office visit of elderly (age ≥65 years) female patients who were diagnosed with osteoporosis/osteopenia [17] and treated at our department or affiliated institutions between January 2015 and September 2019. We have measured serum 25(OH)D levels in osteoporotic patients as a part of the routine pre-treatment evaluation according to our institutional protocol. Patients who were younger than 65, had active endocrine diseases that might have affected serum vitamin D levels, or were missing serum 25(OH)D data were excluded. Additionally, we excluded patients who were taking natural/activated vitamin D as a medication or dietary supplement at the time of the assessment.

### Serum 25(OH)D measurement

Serum 25(OH)D concentrations were evaluated using a chemiluminescent immunoassay method. The serum was separated from the whole blood at the laboratory in our hospital, refrigerated, and transferred to an outside facility. Then, the 25(OH)D level in the serum sample was measured using a LIAISON analyzer (25-OH vitamin D total assay, DiaSorin Liaison, Hitachi Chemical Diagnostics Systems Co., Ltd., Tokyo, Japan).

### Assessment of fall risk, hand grip strength, and other data collection

The fall score introduced by Okochi et al. [18] was used for the fall risk assessment. The scoring system consists of the following questions that estimate tendency of fall in daily activities: (1) Have you fallen in the past 1 year? (2) Do you think that your walking speed has gone down? (3) Do you regularly use a cane? (4) Have you developed a stooped posture? (5) Do you take five or more kinds of medication every day? All five questions were answered with yes or no. Based on the odds ratio for fall, 5 points are added to the score if the patient answers “yes” for question (1), and 2 points each will be added for questions (2)–(5). If the patient answers “no,” no point will be added to the score. The total score ranges from 0 to 13 points, and the patient is considered “high risk” for fall if the score reaches 6 points or above. This cutoff point was described in the original paper by Okochi et al. The risk for fall was four times higher in people with scores ≥6. In addition to the fall score, we administrated a question about anxiety on falling.

As the surrogate marker of systemic muscle strength, the patient-reported dominant-hand grip strength was measured using the Smedley-type hand dynamometer (MY-2080, Matsumiya IkaSeiki Seisakusho Co., Ltd., Tokyo, Japan). Grip strength was measured three times in sitting position, as shown in previous studies [19], and the median value was used in the analyses.

The bone mineral densities of the spine and hip were routinely performed by dual X-ray absorptiometry (Discovery DXA System, Hologic, Inc., Marlborough, MA). Moreover, we administered the self-assessment of dietary calcium intake, which was created and validated for the Japanese population, reflecting the local cultural background [20].

### Data analysis and statistical methods

The comparisons between categorical variables were conducted utilizing the Fisher exact or chi-squared tests. For the comparisons between increased risk and baseline risk groups, the Student’s *t* test was used for the comparisons in normally distributed continuous variables, whereas the Mann–Whitney *U* test was used for the comparisons in non-normally distributed continuous variables. We utilized receiver operating characteristics (ROC) curve analysis for categorical “increased risk” status defined as fall score equal to or over 6 or not to determine the cutoff point of serum 25(OH)D level and grip strength. The cutoff points were defined as the point with the maximum Youden index [21]. Logistic regression analysis with age adjustment was conducted for potential risk factors for fall, such as serum 25(OH)D level and grip strength. The statistical significance level was set at *p* < 0.05. All analyses were conducted in Stat Flex (ver. 7.0.8, Igaku Tokei Kenkyujo Inc., Ube, Japan) as well as R software environment (R ver.3.5.2 GUI).

## Results

**Fig. 1:**
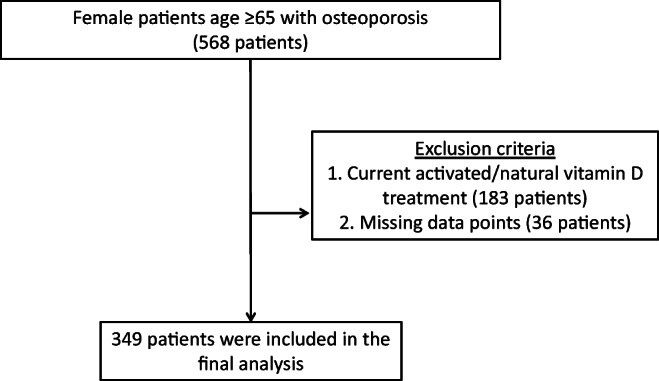
Flow diagram

Of a total of 568 female patients older than 65 years, 183 patients were on activated/natural vitamin D treatment at the first visit, and 36 patients had 1 or more missing data points. After excluding these patients, 349 patients were included in the final analysis (Fig. 1). The median age was 77.0 years (interquartile range, 72.0–84.0 years). The mean serum 25(OH)D level was 15.6 ng/mL (36 nmol/L); approximately 80% of the patients had a 25(OH)D level <20 ng/mL. The baseline demographics of the patients, including laboratory values, bone mineral densities, history of previous fractures, and history of falls, are shown in Table 1.

**Table 1:** Patient demographics

Factor			Overall
*n*			349
Age (years)		Median [IQR]	77.0 [72.0-84.0]
BMI (kg/m2)		Mean (SD)	22.0 (3.75)
(%)	<18.5	*n* (%)	59 (16.9)
	18.5-25	*n* (%)	222 (63.6)
	25-30	*n* (%)	62 (17.8)
	>30	*n* (%)	6 (1.7)
Anti-osteoporotic drugs	Any	*n* (%)	139 (39.8)
	SERM	*n* (%)	13 (3.7)
	Bisphosphonate	*n* (%)	87 (24.9)
	Denosumab	*n* (%)	22 (6.3)
	Teriparatide	*n* (%)	17 (4.9)
Estimated dietary calcium intake (g)		Mean (SD)	13.3 (5.1)
BMD (mg/cm^2^)	Hip	Median [IQR]	0.62 [0.53-0.69]
	Spine	Median [IQR]	0.78 [0.67-0.88]
Previous fracture	Any	*n* (%)	237 (67.9)
	Spine	*n* (%)	226 (64.8)
	Hip	*n* (%)	20 (5.7)
	Pelvis	*n* (%)	9 (2.6)
	Other extremities	*n* (%)	6 (1.7)
Serum calcium (mg/dL)		Mean (SD)	9.4 (0.6)
Creatinine (mg/dL)		Mean (SD)	0.68 (0.29)
eGFR (mL/min/1.73 m^2^)		Mean (SD)	69.6 (20.7)
25(OH)D (ng/mL)		Mean (SD)	15.6 (8.0)
	<20	*n* (%)	278 (79.8)
	20-30	*n* (%)	63 (18.1)
	≥30	*n* (%)	8 (2.3)
History of fall		*n* (%)	126 (36.3)
Apprehension about fall		*n* (%)	190 (54.4)
Grip strength (dominant hand) (kg)		Median [IQR]	15.7 [10.9-20.0]
Fall score		Median [IQR]	6.0 [2.0-9.0]

*IQR* interquartile range; *BMI* body mass index; *SD* standard deviation; *SERM* selective estrogen receptor modulator; *BMD* bone mineral density; *eGFR* estimated glomerular filtration rate; *25(OH)D* 25-hydroxyvitamin D

**Fig. 2:**
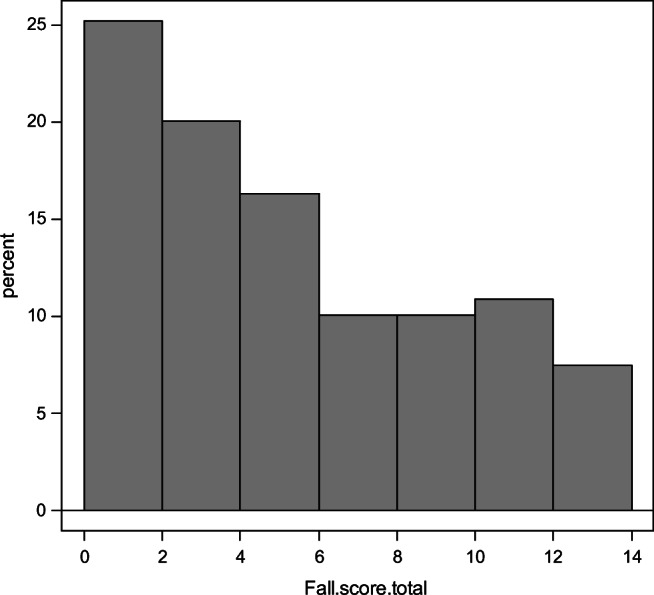
Histogram of fall score distribution

The median fall score was 6.0 (Fig. 2), and 184 patients (52.7%) were considered high risk, with a fall score of 6 or above. In the univariate analysis, the increased risk group was associated with older age, low bone mineral density in the hip, more previous fractures, and low grip strength. Additionally, actual fall and apprehension of fall were more prevalent in the increased risk group. The serum 25(OH)D level was significantly lower in the increased risk group (*p* = 0.011) (Table 2).

**Table 2:** The results of simple comparisons between patients with low and high fall scores

Factor			Fall score <6	Fall score ≥6	*p*-value
*n*			165	184	
Age (years)		Median [IQR]	74.0 [70.0-79.0]	82.0 [76.0-87.0]	<0.001
BMI (kg/m^2^)		Mean (SD)	22.3 (3.5)	21.7 (9.0)	0.139
(%)	<18.5	*n* (%)	23 (13.9)	36 (19.6)	0.282
	18.5-25	*n* (%)	107 (64.8)	115 (62.5)	
	25-30	*n* (%)	31 (18.8)	31 (16.8)	
	35<	*n* (%)	4 (2.4)	2 (1.0)	
Anti-osteoporotic drugs	Any	*n* (%)	68 (41.2)	71 (38.6)	0.662
	SERM	*n* (%)	9 (5.5)	4 (2.2)	0.156
	Bisphosphonate	*n* (%)	42 (25.5)	45 (24.5)	0.901
	Denosumab	*n* (%)	10 (6.1)	12 (6.5)	>0.999
	Teriparatide	*n* (%)	7 (4.2)	10 (5.4)	0.630
Estimated dietary calcium intake (g)		Median [IQR]	13.8 (5.0)	12.9 (5.1)	0.074
BMD (mg/cm^2^)	Hip	Median [IQR]	0.65 [0.58-0.72]	0.58 [0.49-0.67]	<0.001
	Spine	median [IQR]	0.78 [0.68-0.88]	0.78 [0.65-0.88]	0.684
Previous fracture	Any	*n* (%)	96 (58.2)	141 (76.6)	<0.001
	Spine	*n* (%)	93 (56.4)	133 (72.3)	0.002
	Hip	*n* (%)	4 (2.4)	16 (8.7)	0.019
Serum calcium (mg/dL)		Mean (SD)	9.4 (0.7)	9.5 (0.5)	0.104
eGFR (mL/min/1.73 m^2^)		Mean (SD)	71.5 (17.1)	67.8 (23.4)	0.097
25(OH)D (ng/mL)		Mean (SD)	16.7 (6.8)	14.6 (8.8)	0.011
Grip strength (kg)		Median [IQR]	18.00 [15.00-21.20]	13.00 [9.30, 17.10]	<0.001
History of fall		*n* (%)	7 (4.3)	119 (65.0)	<0.001
Apprehension about fall		*n* (%)	56 (33.9)	134 (72.8)	<0.001

*IQR* interquartile range; *BMI* body mass index; *SD* standard deviation; *SERM* selective estrogen receptor modulator; *BMD* bone mineral density; *eGFR* estimated glomerular filtration rate; *25(OH)D* 25-hydroxyvitamin D

**Fig. 3:**
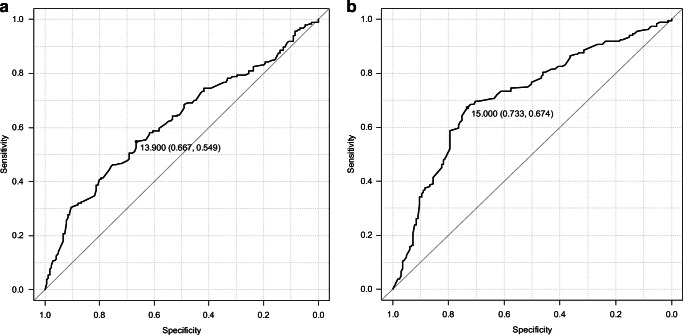
The results of receiver operating characteristic curve analysis for identification of patients with fall score ≥6. **a** Serum 25(OH)D level. **b** Hand grip strength in the dominant hand

The area under the ROC curve (AUC) of serum 25(OH)D was 0.62 (95% confidential interval (CI) 0.56–0.68) and the AUC of grip strength was 0.71 (CI, 0.66–0.77). Based on the ROC curve analyses, the cutoff values for increased fall risk, specifically in osteoporotic elderly women, were 14 ng/mL (35 nmol/L) in serum 25(OH)D level and 15 kg in dominant-hand grip (Fig. 3). An age-adjusted multivariate analysis was conducted. Low serum 25(OH)D level and low grip strength were independent risk factors for a high fall risk score (Table 3).

**Table 3:** The results of age-adjusted logistic regression analysis

Factor	Odds ratio (95% CI) for high fall risk score	*p*-value
Serum 25(OH)D <14 ng/dL	1.67 (1.02-2.70)	0.041
Hand grip strength <15 kg	2.86 (1.67-4.76)	<0.001

*CI* confidence interval

**Fig. 4:**
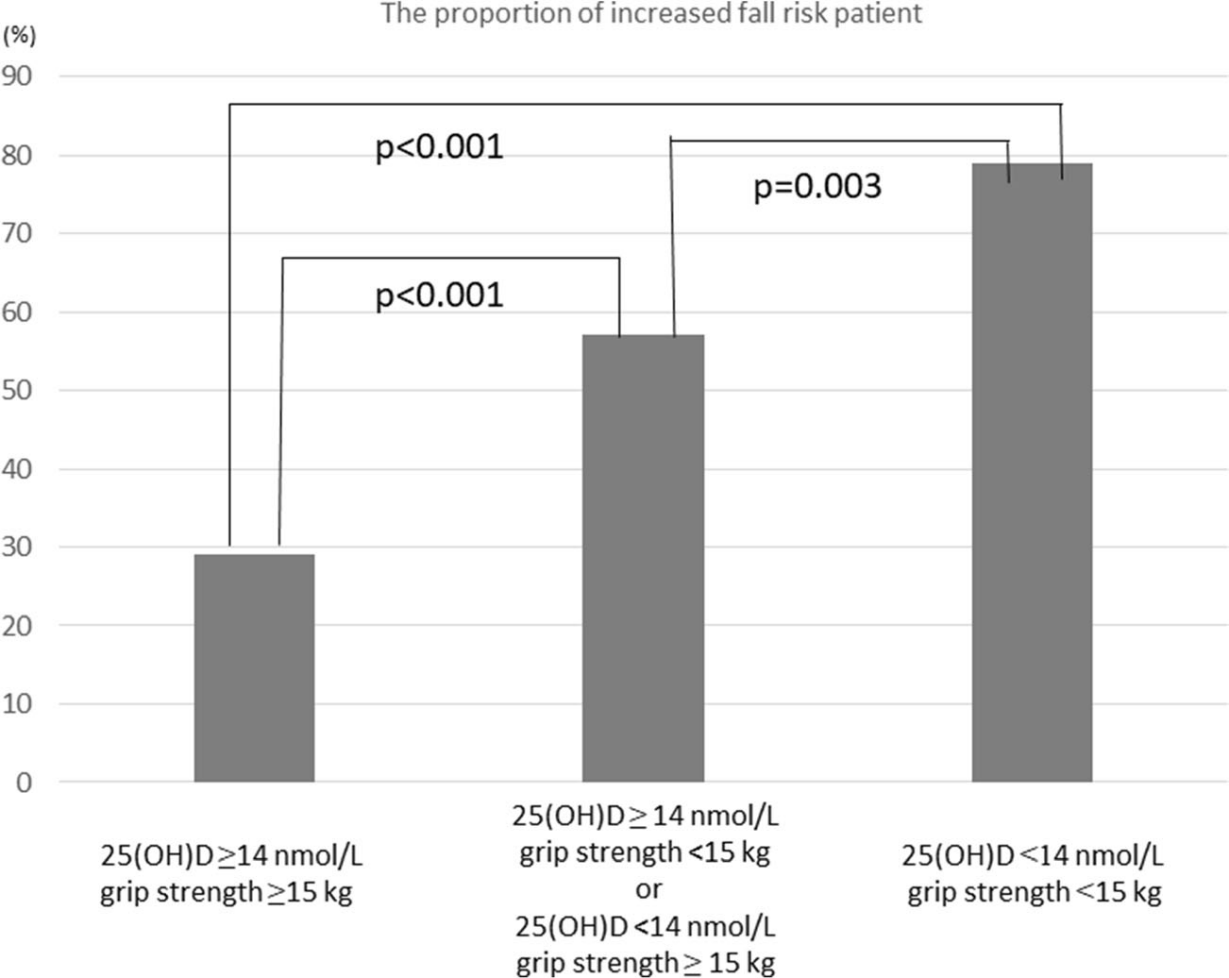
The association of the proportion of patients with increased fall risks (fall score ≥6) and the categorized 25(OH)D and grip strength

As an ad hoc analysis, we investigated the association between the proportion of patients with increased fall risks whose fall score was ≥6 and two factors using the cutoff points (25(OH)D, 14 ng/dL; grip strength, 15 kg). In patients with 25(OH)D ≥14 ng/dL, grip strength ≥15 kg, the proportion of fall score ≥6 patients was 29.0%, whereas in patients with 25(OH)D <14 nmol/L, grip strength <15 kg, the proportion was 78.9% (*p* < 0.001) (Fig. 4). In addition, the age-adjusted logistic regression analysis showed a linear increase in the odds ratio with the number of risk factors (1 risk factor; odds ratio (95% CI) 2.31 (1.34–3.99), *p* = 0.003; 2 risk factors 4.43 (2.21-8.89), *p* <0.001).

## Discussion

In this study, we proposed that the cutoff of serum 25(OH)D level specific for identification of increased risk group in elderly osteoporotic women was 14 mg/dL (35 nmol/L) and that low grip strength was associated with increased fall risk score. Both effects were mutually independent and independent of age.

The current normal value for serum 25(OH)D in the guidelines is based on studies regarding fractures. Studies have demonstrated that the target levels of 25(OH)D for non-vertebral fracture prevention were 21.6–39.6 ng/mL (54–99 nmol/L) [22]. Reportedly, there are regional differences of serum vitamin D level in the general and elderly population. According to the IOF position statement in 2010, the proportion of vitamin D deficiency is higher in Eastern Asian countries than in European and North American countries [10]. In our study, approximately 80% of our patients were categorized as vitamin D deficient. Although these target levels are useful in guiding vitamin D treatment and nutritional support for fracture prevention, it is difficult to use the current cutoff values for further stratification of fall risk in osteoporotic elderly women because of the abovementioned reasons. Our results suggest that a 25(OH)D level under 14 ng/mL (35 nmol/L) can be considered the cutoff point for identifying the increased risk group in osteoporotic elderly female patients*.*

We utilized the simple fall screening tool by Okochi et al. for fall risk assessment [18]. This is an interview-based scoring system that contains five questions and ranges from 0 (no risk) to 13 (high risk). In their original study, 28% of patients with a score of 6 points or higher experienced at least one fall within the next 6 months, whereas only 7% of patients with a score of less than 6 reported a fall. The authors concluded that intervention for fall prevention was recommended for patients with a score of less than 6. Currently, various fall risk assessment tools are available [23–25]. The Morse Fall Scale (MFS) [26] is a commonly used fall score. The MFS was originally developed and has been used to evaluate the fall risk for in-hospital patients. In contrast, our study participants were community-dwelling people in the outpatient clinic. Some items in the MFS, such as IV/heparin lock, are not applicable in outpatient settings. Furthermore, we chose the fall scale because this scale was invented and validated in the patients’ mother language (Japanese) and there was no cross-cultural adaptation issue. Although we did not directly compare multiple scoring systems in this study and there has been no study to directly compare the predictive value of this assessment system with other questionnaire-based assessment systems, the Okochi fall scores were significantly associated with a history of fall as well as the subjective apprehension of fall, and these results suggest the usefulness of this assessment system in our patient population. Previous studies have reported various muscular functional assessment methods for fall risk evaluation. Although it is still controversial [27–30], grip strength measurement is one of the common surrogate markers for overall systemic muscle strength [30] and is probably the easiest method of assessing muscle function. For fall risk assessment, previous studies have demonstrated that low grip strength was a risk factor for fall in various patient populations, such as patients who had undergone lumbar spine surgery [15] and patients with a history of chronic liver disease [31]. In addition, one previous meta-analysis showed that upper extremity weakness, including low hand grip strength, was associated with a higher risk of fall [32]. Conversely, it has been reported that lower extremity strength, such as knee extension strength, is more predictive for fall risk assessment. In the abovementioned meta-analysis, lower extremity assessments showed a higher odds ratio than upper extremity assessments [32]. However, lower extremity muscle strength testing usually requires more time than simple grip strength testing. Given the high predictive value in our study, hand grip strength testing can be routinely used for fall risk assessment in clinical practice.

Moreover, our study demonstrated the additive effect of serum 25(OH)D level and grip strength. Interestingly, these effects were mutually independent and independent of age. Vitamin D receptor- and vitamin D-metabolizing enzymes are distributed in almost all organ systems. The effect of vitamin D on bone health has been established, whereas there has been less studies about extra-skeletal effects of vitamin D on muscles and balance [33]. In addition to muscle strength, vitamin D has effects on locomotor coordination and balance, which are related to lower fall risk scores. Further studies are required to clarify these points. Although the background mechanism is largely yet to be determined, our results can be applied in daily practice to evaluate fall risk in elderly female patients.

There were several limitations to this study. First, our study population consisted of elderly Japanese women who were visiting an osteoporosis clinic. Our results might not be applicable to other geographical/ethnic populations. The generalizability of these results should be confirmed in various patient populations. Although patients undergoing vitamin D treatment were excluded, 25(OH)D levels are affected by the intake of natural vitamin D from food and ultraviolet rays. The information about dietary intake of vitamin D, duration of sunlight exposure, daytime activity, and UV protection was not included. These issues should be addressed in future studies. We utilized the fall risk score by Okochi et al. since it was developed for the outpatient setting and validated in the patients’ mother language; however, we did not utilize other fall risk assessment systems. Finally, we investigated the fall risk in elderly osteoporotic women, in whom the baseline fall risk is likely higher as the baseline. The cutoff values cannot be applied for fall risk assessment in the general population or other age groups. For instance, since certain fragile fractures are associated with fall, such as the incidence of distal radius fracture increases before the common onset age of osteoporosis (33), the cutoff value of increased fall risk in 25(OH)D for the younger population, such as people in their early 50s, is likely higher than 14 mg/dL and even possibly higher than the aimed value for osteoporosis treatment. Further studies are required to address these issues.

In conclusion, our results demonstrated that both low serum 25(OH)D level and low grip strength were independently associated with increased fall risk among elderly female Japanese patients with osteoporosis. The serum 25(OH)D and grip strength cutoff values specific to increased fall risk in this patient population were 14 mg/dL (35 nmol/L) and 15 kg, respectively. These findings might be used for further risk stratification of this patient group, in which the baseline risk for fall is higher and potential impact of fall is greater than that of the general population.
